# Supplementary material for: IDR2D identifies reproducible genomic interactions
Source: Nucleic Acids Res. 2020 Feb 3;48(6):e31. doi: 10.1093/nar/gkaa030 (PMC7102997; doi:10.1093/nar/gkaa030)
Supplement: gkaa030_Supplemental_File [file gkaa030_supplemental_file.pdf]

Supplementary Material

IDR2D identifies reproducible genomic interactions

Konstantin Krismer[1,2], Yuchun Guo[1] and David K. Gifford[1,2,3,*]

[1]Computer Science and Artificial Intelligence Laboratory, Massachusetts Institute of Technology, 32 Vassar Street, Cambridge, MA 02139, USA
[2]Department of Biological Engineering, Massachusetts Institute of Technology, 77 Massachusetts Avenue, Cambridge, MA 02139, USA
[3]Department of Electrical Engineering and Computer Science, Massachusetts Institute of Technology, 77 Massachusetts Avenue, Cambridge, MA 02139, USA

1 IDR2D procedure

We first define $\Omega_{x_{i,1}}$ as the set of interactions in replicate 2 that overlaps with the interaction $x_{i,1}$ in replicate 1. Two interactions $x_{i,1}$ and $x_{j,2}$ are overlapping, if both of their interaction anchors are overlapping or are less than a predefined maximum gap away from each other.

If interaction $x_{i,1}$ overlaps with more than one interaction in replicate 2, the ambiguous mapping is resolved by choosing $x_{*,2}$ in the following way:

$$x_{*,2} = \underset{x_{j,2} \in \Omega_{x_{i,1}}}{\operatorname{argmin}} f(x_{i,1}, x_{j,2}), \tag{1}$$

where $f(\cdot,\cdot)$ is the *ambiguity resolution value* (ARV) between an interaction in replicate 1 and an overlapping interaction in replicate 2. The three supported ambiguity resolution methods are depicted in figure 1D of the main manuscript.

To estimate irreproducible discovery rates, IDR2D uses a two-component copula mixture model that is identical to the model described for the original IDR method, with one component for irreproducible interaction pairs and another one for reproducible interaction pairs. After a bijective mapping between interactions in replicate 1 and interactions in replicate 2 is established, the posterior probability that an interaction pair i denoted as $(x_{i,1}, x_{i,2})$, with significance values from replicates 1 and 2 belongs to the irreproducible component is defined as

$$\Pr((x_{i,1}, x_{i,2}); \theta_{\text{irrep}}, \theta_{\text{rep}}) = \frac{C_{\text{irrep}}}{C_{\text{irrep}} + C_{\text{reproducible}}} \tag{2}$$

$$C_{\text{irrep}} = \pi_0 h_0 \left(G^{-1}\left(F_1\left(x_{i,1}\right)\right), G^{-1}\left(F_2\left(x_{i,2}\right)\right)\right) \tag{3}$$

$$C_{\text{rep}} = \pi_1 h_1 \left(G^{-1}\left(F_1\left(x_{i,1}\right)\right), G^{-1}\left(F_2\left(x_{i,2}\right)\right)\right) \tag{4}$$

$$h_0 \sim \mathcal{N}\left(\begin{pmatrix}0\\0\end{pmatrix}, \begin{pmatrix}1 & 0\\0 & 1\end{pmatrix}\right) \tag{5}$$

$$h_1 \sim \mathcal{N}\left(\begin{pmatrix}\mu_1\\\mu_1\end{pmatrix}, \begin{pmatrix}\sigma_1^2 & \rho_1\sigma_1^2\\\rho_1\sigma_1^2 & \sigma_1^2\end{pmatrix}\right), \tag{6}$$

where $\theta_{\text{irrep}} = \pi_0$ and $\theta_{\text{rep}} = (\pi_1, \mu_1, \sigma_1^2, \rho_1)$, the estimated parameters of the two components (see Li et al. [1] section 2.2.3 for details on the estimation procedure). For a definition of G, and F_1 and F_2, the marginal distributions of the coordinates in the two replicates, see Li et al. [1] section 2.2.2 for details.

*To whom correspondence should be addressed. Tel: +1 617 253 6039; Email: gifford@mit.edu

2 Supplementary Figures

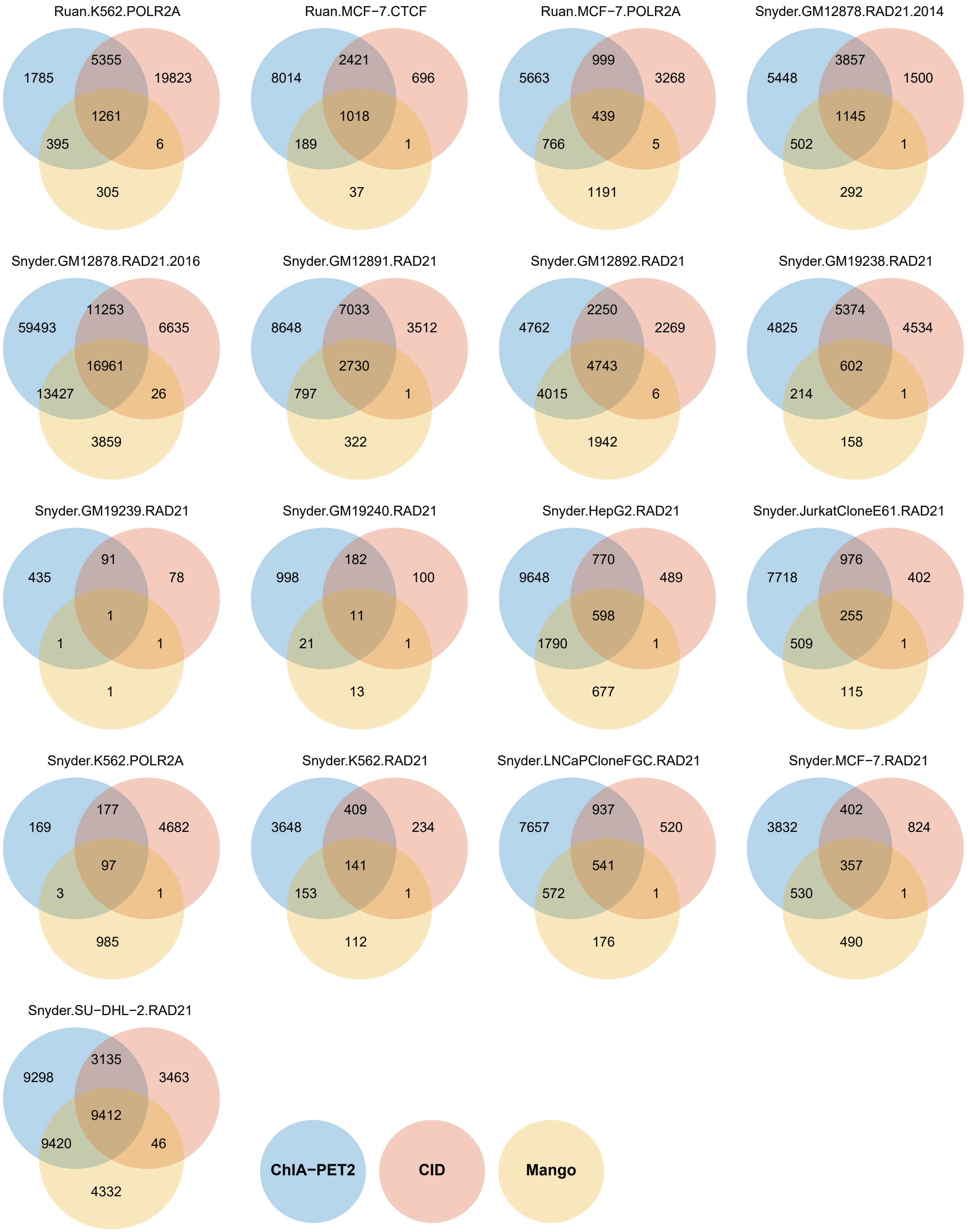

Supplementary Figure S1: Overlap between reproducible interactions identified by ChIA-PET2, CID, and Mango, and IDR2D. Each Venn diagram shows the overlap between interactions called by ChIA-PET2, CID, and Mango that had an IDR of less than 0.05 of one of 17 replicated ENCODE ChIA-PET experiments.

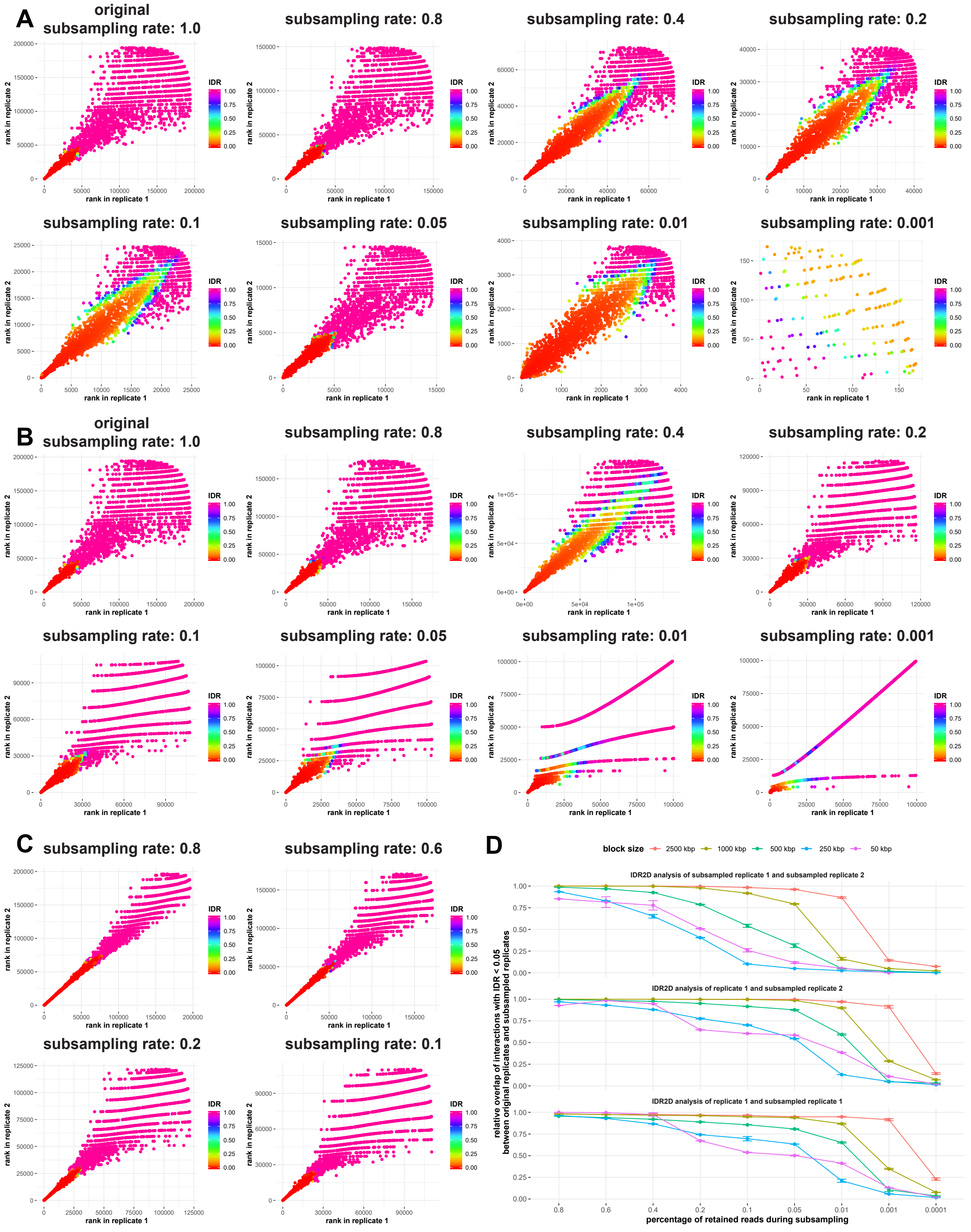

Supplementary Figure S2: Influence of sequencing depth on IDR2D analysis. All panels are based on IDR2D analysis of subsampled replicates of GSE63525, chromosome 16. Panels **A** - **C** use block sizes of 50 kbp. (**A**) Rank scatterplots of IDR2D analysis of subsampled replicate 1 and subsampled replicate 2 at read retention rates of 1.0 (all reads retained) to 0.001 (99.9% of reads removed). (**B**) Rank scatterplots of IDR2D analysis of original replicate 1 and subsampled replicate 2. (**C**) Rank scatterplots of IDR2D analysis of original replicate 1 and subsampled replicate 1. (**D**) Relative overlap of interactions with IDR < 0.05 between subsampled and original IDR2D analysis at various read retention rates and block sizes.

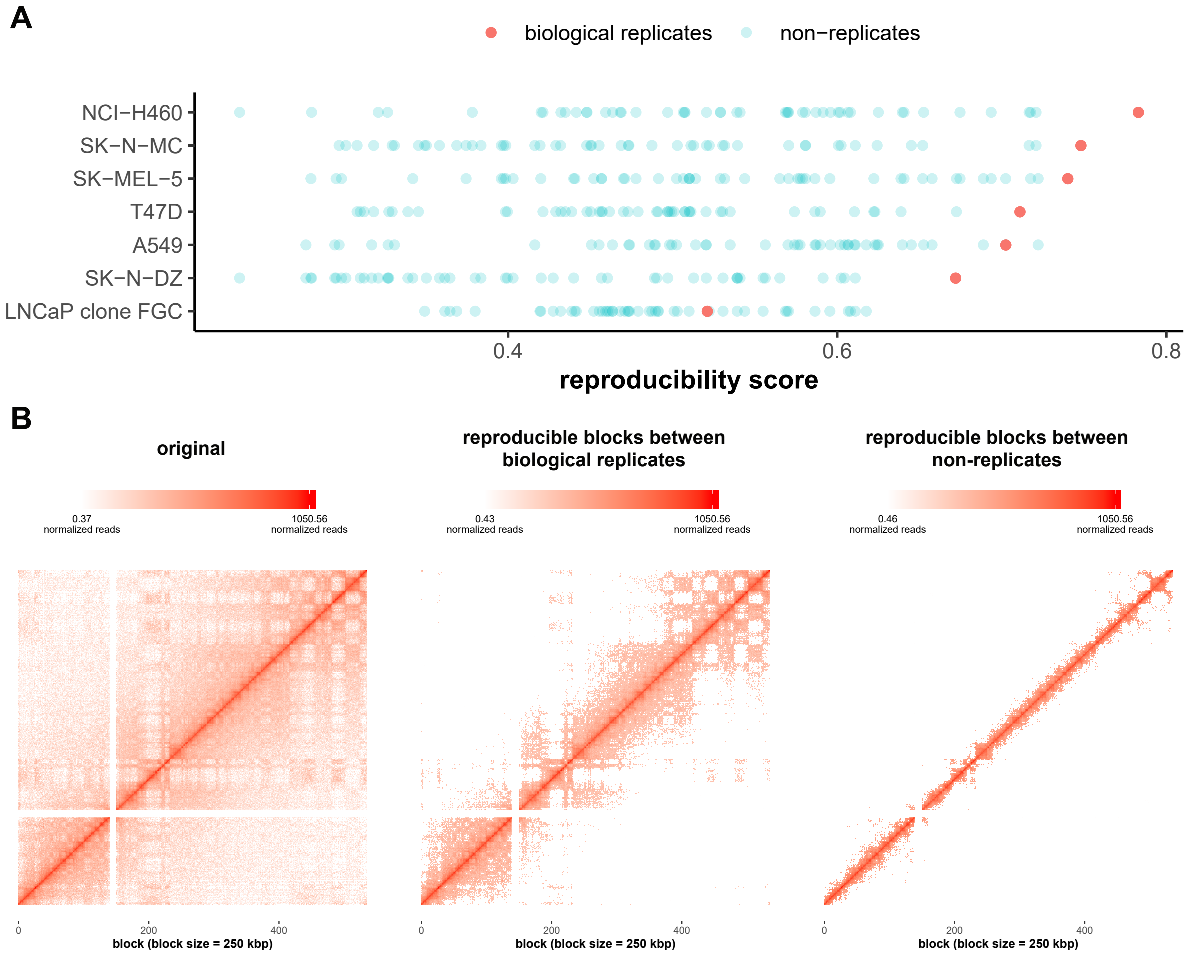

Supplementary Figure S3: Hi-C reproducibility between replicates and non-replicates. (**A**) Relative reproducibility scores of IDR2D analyses between biological replicates and non-replicates. Scores were calculated based on the number of highly reproducible blocks (IDR < 0.01) in the contact matrix, summarizing results of all chromosomes and averaged over various resolutions (block sizes of 2.5 Mbp, 1 Mbp, 500 kbp, 250 kbp, 100 kbp, 50 kbp, 25 kbp, 10 kbp, and 5 kbp). (**B**) Visualizations of contact maps of chromosome 12 showing all blocks (left), blocks with IDR < 0.05 between two biological replicates in cell line NCI-H460 (middle), and blocks with IDR < 0.05 between two non-replicates (right).

3 Supplementary Tables

ChIA-PET dataset identifier	Target	Cell line	ENCODE identifier
Ruan.K562.POLR2A	POLR2A	K562	ENCSR000BZY
Ruan.MCF-7.POLR2A	POLR2A	MCF-7	ENCSR000CAA
Snyder.K562.POLR2A	POLR2A	K562	ENCSR000FDC
Ruan.MCF-7.CTCF	CTCF	MCF-7	ENCSR000CAD
Snyder.GM12878.RAD21.2014	RAD21	GM12878	ENCSR752QCX
Snyder.GM12878.RAD21.2016	RAD21	GM12878	ENCSR981FNA
Snyder.GM12891.RAD21	RAD21	GM12891	ENCSR299VMZ
Snyder.GM12892.RAD21	RAD21	GM12892	ENCSR033GUP
Snyder.GM19238.RAD21	RAD21	GM19238	ENCSR527RXH
Snyder.GM19239.RAD21	RAD21	GM19239	ENCSR479MTN
Snyder.GM19240.RAD21	RAD21	GM19240	ENCSR312TUD
Snyder.HepG2.RAD21	RAD21	HepG2	ENCSR014ZXR
Snyder.JurkatCloneE61.RAD21	RAD21	Jurkat clone E61	ENCSR465NNU
Snyder.K562.RAD21	RAD21	K562	ENCSR000FDB
Snyder.LNCaPCloneFGC.RAD21	RAD21	LNCaP clone FGC	ENCSR011ITK
Snyder.MCF-7.RAD21	RAD21	MCF-7	ENCSR716WZI
Snyder.SU-DHL-2.RAD21	RAD21	SU-DHL-2	ENCSR466AXT
**HiChIP dataset identifier**	**Target**	**Cell line**	**GEO identifier**
Chang.GM12878.H3K27ac	H3K27ac	GM12878	GSE101498
Chang.K562.H3K27ac	H3K27ac	K562	GSE101498
Chang.mES.H3K27ac	H3K27ac	mES	GSE101498
Chang.MyLa.H3K27ac	H3K27ac	MyLa	GSE101498
Chang.Naive.CTCF	CTCF	Naive	GSE101498
Chang.Naive.H3K27ac	H3K27ac	Naive	GSE101498
Chang.Th17.H3K27ac	H3K27ac	Th17	GSE101498
Chang.Treg.H3K27ac	H3K27ac	Treg	GSE101498
Flynn.GM12878.Smc1a	Smc1a	GM12878	GSE80820
**HiC dataset identifier**		**Cell line**	**GEO identifier**
Lieberman.GM12878		GM12878	GSE63525
Lieberman.Patski		Patski	GSE71831
Skok.NSD2		multiple myeloma	GSE131651
**HiC dataset identifier and cell line**			**ENCODE identifier**
A549			ENCSR444WCZ
SK-N-DZ			ENCSR105KFX
SK-MEL-5			ENCSR312KHQ
LNCaP clone FGC			ENCSR346DCU
NCI-H460			ENCSR489OCU
T47D			ENCSR549MGQ
SK-N-MC			ENCSR834DXR

Supplementary Table S1: ChIA-PET, HiChIP, and HiC datasets used in this study.

Dataset identifier	Total int.	Rep. int.	IDR < 0.05	IDR < 0.01
Ruan.K562.POLR2A	15,029	9594	8816	4919
Ruan.MCF-7.POLR2A	23,540	14,718	2714	1251
Snyder.K562.POLR2A	20,683	9142	446	220
Ruan.MCF-7.CTCF	42,958	12,042	11,664	10,503
Snyder.GM12878.RAD21.2014	26,376	11,176	10,977	10,191
Snyder.GM12878.RAD21.2016	354,536	127,172	101,330	11,164
Snyder.GM12891.RAD21	26,180	20,144	19,245	14,628
Snyder.GM12892.RAD21	33,731	16,253	15,906	14,401
Snyder.GM19238.RAD21	90,887	12,471	11,031	1676
Snyder.GM19239.RAD21	50,387	1712	526	47
Snyder.GM19240.RAD21	6225	2226	1212	94
Snyder.HepG2.RAD21	47,544	18,308	12,819	1300
Snyder.JurkatCloneE61.RAD21	18,408	9745	889	367
Snyder.K562.RAD21	5540	4470	4352	3782
Snyder.LNCaPCloneFGC.RAD21	34,260	12,456	9718	1056
Snyder.MCF-7.RAD21	109,485	14,793	5117	639
Snyder.SU-DHL-2.RAD21	99,864	39,891	31,437	3266

Supplementary Table S2: IDR2D analysis of ChIA-PET interactions called by ChIA-PET2. Columns are (1) *total number of interactions in replicate 1*, (2) *number of reproducible interactions*, (3) *number of reproducible interactions with IDR* < 0.05, and (4) *number of reproducible interactions with IDR* < 0.01.

Dataset identifier	Total int.	Rep. int.	IDR < 0.05	IDR < 0.01
Ruan.K562.POLR2A	116,932	40,693	26,958	8698
Ruan.MCF-7.POLR2A	98,484	33,147	5064	681
Snyder.K562.POLR2A	51,323	12,352	6300	1513
Ruan.MCF-7.CTCF	53,351	14,321	4163	1162
Snyder.GM12878.RAD21.2014	49,102	16,309	6556	1733
Snyder.GM12878.RAD21.2016	394,045	105,704	36,662	10,363
Snyder.GM12891.RAD21	45,295	29,509	13,398	3633
Snyder.GM12892.RAD21	70,222	26,637	9594	2457
Snyder.GM19238.RAD21	162,936	23,444	10,573	2745
Snyder.GM19239.RAD21	98,449	4074	169	22
Snyder.GM19240.RAD21	15,473	4325	295	65
Snyder.HepG2.RAD21	53,725	16,886	1876	267
Snyder.JurkatCloneE61.RAD21	46,965	14,837	1642	228
Snyder.K562.RAD21	13,891	8791	790	104
Snyder.LNCaPCloneFGC.RAD21	66,356	19,021	2013	299
Snyder.MCF-7.RAD21	43,930	14,754	1641	262
Snyder.SU-DHL-2.RAD21	132,610	43,197	17,460	4765

Supplementary Table S3: IDR2D analysis of ChIA-PET interactions called by CID. Columns are (1) *total number of interactions in replicate 1*, (2) *number of reproducible interactions*, (3) *number of reproducible interactions with IDR* < 0.05, and (4) *number of reproducible interactions with IDR* < 0.01.

Dataset identifier	Total int.	Rep. int.	IDR < 0.05	IDR < 0.01
Ruan.K562.POLR2A	16,847	10,581	2273	1149
Ruan.MCF-7.POLR2A	23,540	14,718	2714	1251
Snyder.K562.POLR2A	17,191	7051	2419	477
Ruan.MCF-7.CTCF	41,542	11,975	1274	502
Snyder.GM12878.RAD21.2014	29,770	12,057	1993	619
Snyder.GM12878.RAD21.2016	166,543	69,897	36,597	8039
Snyder.GM12891.RAD21	28,426	21,983	3976	338
Snyder.GM12892.RAD21	37,730	17,900	11,050	1182
Snyder.GM19238.RAD21	97,679	13,084	1038	109
Snyder.GM19239.RAD21	57,254	2231	1	0
Snyder.GM19240.RAD21	6462	2219	47	14
Snyder.HepG2.RAD21	26,747	12,285	3091	494
Snyder.JurkatCloneE61.RAD21	18,408	9745	889	367
Snyder.K562.RAD21	7203	5474	412	98
Snyder.LNCaPCloneFGC.RAD21	24,065	11,992	1308	420
Snyder.MCF-7.RAD21	20,277	10,412	1453	511
Snyder.SU-DHL-2.RAD21	71,043	34,226	24,909	2552

Supplementary Table S4: IDR2D analysis of ChIA-PET interactions called by Mango. Columns are (1) *total number of interactions in replicate 1*, (2) *number of reproducible interactions*, (3) *number of reproducible interactions with IDR* < 0.05, and (4) *number of reproducible interactions with IDR* < 0.01.

Dataset identifier	Total int.	Rep. int.	IDR < 0.05	IDR < 0.01
Chang.GM12878.H3K27ac	8,170,291	4,076,304	2,133,248	408,385
Chang.K562.H3K27ac	6,059,672	2,724,718	991,394	295,193
Chang.mES.H3K27ac	4,997,523	2,593,482	1,902,221	118,607
Chang.MyLa.H3K27ac	6,010,073	2,745,081	1,052,481	345,503
Chang.Naive.CTCF	1,368,563	52,845	6885	1607
Chang.Naive.H3K27ac	69,861	49,823	13,504	1821
Chang.Th17.H3K27ac	2,135,349	1,418,902	889,736	188,608
Chang.Treg.H3K27ac	365,827	253,529	105,227	15,856
Flynn.GM12878.Smc1a	6,036,994	2,232,884	369,344	4166

Supplementary Table S5: IDR2D analysis of HiChIP interactions called by CID. Columns are (1) *total number of interactions in replicate 1*, (2) *number of reproducible interactions*, (3) *number of reproducible interactions with IDR* < 0.05, and (4) *number of reproducible interactions with IDR* < 0.01.

Dataset identifier	Total int.	Rep. int.	IDR < 0.05	IDR < 0.01
Flynn.GM12878.Smc1a	2,312,685	1,126,785	71,850	2892

Supplementary Table S6: IDR2D analysis of HiChIP interactions called by hichipper. Columns are (1) *total number of interactions in replicate 1*, (2) *number of reproducible interactions*, (3) *number of reproducible interactions with IDR* < 0.05, and (4) *number of reproducible interactions with IDR* < 0.01.

Dataset identifier	Chr.	Resolution	Non-empty blocks	IDR < 0.05	IDR < 0.01
Lieberman.GM12878	chr1	5,000	810,663	195,113	180,896
Lieberman.GM12878	chr1	10,000	808,144	222,766	210,847
Lieberman.GM12878	chr1	25,000	531,624	179,436	173,794
Lieberman.GM12878	chr1	50,000	491,974	112,121	110,096
Lieberman.GM12878	chr1	100,000	629,037	141,128	130,959
Lieberman.GM12878	chr1	250,000	323,444	151,273	145,581
Lieberman.GM12878	chr1	500,000	100,553	84,852	76,715
Lieberman.GM12878	chr1	1,000,000	26,188	25,470	24,582
Lieberman.GM12878	chr1	2,500,000	4,278	4,058	3,893
Lieberman.GM12878	chr2	5,000	723,520	196,615	56,908
Lieberman.GM12878	chr2	10,000	782,056	272,553	92,570
Lieberman.GM12878	chr2	25,000	554,758	182,181	176,264
Lieberman.GM12878	chr2	50,000	497,542	193,676	112,630
Lieberman.GM12878	chr2	100,000	679,578	155,472	141,249
Lieberman.GM12878	chr2	250,000	373,283	230,169	188,254
Lieberman.GM12878	chr2	500,000	113,927	89,447	79,260
Lieberman.GM12878	chr2	1,000,000	29,243	28,656	27,907
Lieberman.GM12878	chr2	2,500,000	4,753	4,672	4,591
Lieberman.GM12878	chr3	5,000	599,232	135,122	127,995
Lieberman.GM12878	chr3	10,000	640,635	168,021	159,080
Lieberman.GM12878	chr3	25,000	461,102	232,605	156,520
Lieberman.GM12878	chr3	50,000	403,388	168,187	113,571
Lieberman.GM12878	chr3	100,000	549,425	139,110	41,564
Lieberman.GM12878	chr3	250,000	277,284	151,653	143,743
Lieberman.GM12878	chr3	500,000	76,208	67,754	59,398
Lieberman.GM12878	chr3	1,000,000	19,306	19,074	18,766
Lieberman.GM12878	chr3	2,500,000	3,240	3,233	3,225
Lieberman.GM12878	chr4	5,000	420,874	121,508	34,313
Lieberman.GM12878	chr4	10,000	485,037	118,935	112,651
Lieberman.GM12878	chr4	25,000	390,484	187,368	105,089
Lieberman.GM12878	chr4	50,000	325,098	89,002	87,087
Lieberman.GM12878	chr4	100,000	469,066	89,047	84,236
Lieberman.GM12878	chr4	250,000	219,566	131,924	125,304
Lieberman.GM12878	chr4	500,000	70,476	51,295	49,639
Lieberman.GM12878	chr4	1,000,000	17,960	15,055	14,515
Lieberman.GM12878	chr4	2,500,000	2,926	2,702	2,611
Lieberman.GM12878	chr5	5,000	493,042	137,013	39,869
Lieberman.GM12878	chr5	10,000	543,723	190,010	65,579
Lieberman.GM12878	chr5	25,000	405,801	204,970	134,614
Lieberman.GM12878	chr5	50,000	369,247	87,537	85,909
Lieberman.GM12878	chr5	100,000	471,544	104,213	97,556
Lieberman.GM12878	chr5	250,000	206,777	118,590	113,362
Lieberman.GM12878	chr5	500,000	62,465	46,734	44,794

Continued on next page

Supplementary Table S7 – *Continued from previous page*

**Dataset identifier**	**Chr.**	**Resolution**	**Non-empty blocks**	**IDR < 0.05**	**IDR < 0.01**
Lieberman.GM12878	chr5	1,000,000	15,934	14,957	14,030
Lieberman.GM12878	chr5	2,500,000	2,701	2,661	2,622
Lieberman.GM12878	chr6	5,000	533,326	144,106	41,979
Lieberman.GM12878	chr6	10,000	559,146	148,886	141,479
Lieberman.GM12878	chr6	25,000	391,304	203,798	146,938
Lieberman.GM12878	chr6	50,000	335,017	85,056	83,452
Lieberman.GM12878	chr6	100,000	450,897	90,657	85,894
Lieberman.GM12878	chr6	250,000	205,011	115,894	109,533
Lieberman.GM12878	chr6	500,000	56,749	53,852	50,613
Lieberman.GM12878	chr6	1,000,000	14,345	14,267	14,159
Lieberman.GM12878	chr6	2,500,000	2,415	2,401	2,384
Lieberman.GM12878	chr7	5,000	437,592	117,112	32,835
Lieberman.GM12878	chr7	10,000	472,781	162,683	54,365
Lieberman.GM12878	chr7	25,000	346,205	171,321	112,967
Lieberman.GM12878	chr7	50,000	309,624	122,117	67,032
Lieberman.GM12878	chr7	100,000	398,266	89,716	82,787
Lieberman.GM12878	chr7	250,000	172,975	90,090	86,290
Lieberman.GM12878	chr7	500,000	48,503	45,505	41,819
Lieberman.GM12878	chr7	1,000,000	12,445	12,341	12,186
Lieberman.GM12878	chr7	2,500,000	2,080	2,043	2,013
Lieberman.GM12878	chr8	5,000	403,695	110,526	31,924
Lieberman.GM12878	chr8	10,000	443,115	113,894	107,971
Lieberman.GM12878	chr8	25,000	335,194	111,901	107,842
Lieberman.GM12878	chr8	50,000	313,846	72,524	71,048
Lieberman.GM12878	chr8	100,000	392,026	91,831	85,530
Lieberman.GM12878	chr8	250,000	149,140	99,873	93,884
Lieberman.GM12878	chr8	500,000	40,951	38,507	36,229
Lieberman.GM12878	chr8	1,000,000	10,510	10,439	10,314
Lieberman.GM12878	chr8	2,500,000	1,770	1,763	1,755
Lieberman.GM12878	chr9	5,000	360,162	93,962	25,567
Lieberman.GM12878	chr9	10,000	372,945	100,735	94,393
Lieberman.GM12878	chr9	25,000	268,070	84,515	81,627
Lieberman.GM12878	chr9	50,000	230,751	92,595	45,628
Lieberman.GM12878	chr9	100,000	251,298	58,406	55,179
Lieberman.GM12878	chr9	250,000	88,293	52,796	50,496
Lieberman.GM12878	chr9	500,000	25,407	23,488	22,560
Lieberman.GM12878	chr9	1,000,000	6,969	6,609	6,493
Lieberman.GM12878	chr9	2,500,000	1,265	1,227	1,212
Lieberman.GM12878	chr10	5,000	427,941	115,314	33,459
Lieberman.GM12878	chr10	10,000	455,285	160,620	54,640
Lieberman.GM12878	chr10	25,000	322,398	108,634	104,978
Lieberman.GM12878	chr10	50,000	309,327	105,336	47,690

Continued on next page

Supplementary Table S7 – *Continued from previous page*

Dataset identifier	Chr.	Resolution	Non-empty blocks	IDR < 0.05	IDR < 0.01
Lieberman.GM12878	chr10	100,000	361,011	91,350	84,114
Lieberman.GM12878	chr10	250,000	129,813	96,705	83,458
Lieberman.GM12878	chr10	500,000	35,256	34,572	33,536
Lieberman.GM12878	chr10	1,000,000	9,001	8,853	8,647
Lieberman.GM12878	chr10	2,500,000	1,540	1,488	1,439
Lieberman.GM12878	chr11	5,000	484,598	118,233	109,147
Lieberman.GM12878	chr11	10,000	469,228	131,318	124,348
Lieberman.GM12878	chr11	25,000	328,871	101,285	98,269
Lieberman.GM12878	chr11	50,000	335,391	72,504	70,876
Lieberman.GM12878	chr11	100,000	372,469	100,819	92,610
Lieberman.GM12878	chr11	250,000	131,103	95,125	81,196
Lieberman.GM12878	chr11	500,000	35,087	34,344	33,245
Lieberman.GM12878	chr11	1,000,000	8,909	8,890	8,860
Lieberman.GM12878	chr11	2,500,000	1,485	1,466	1,451
Lieberman.GM12878	chr12	5,000	452,496	119,260	34,355
Lieberman.GM12878	chr12	10,000	459,416	126,364	119,449
Lieberman.GM12878	chr12	25,000	326,782	162,196	109,901
Lieberman.GM12878	chr12	50,000	275,015	116,548	75,840
Lieberman.GM12878	chr12	100,000	330,069	91,506	28,598
Lieberman.GM12878	chr12	250,000	129,056	91,009	84,005
Lieberman.GM12878	chr12	500,000	34,631	33,567	32,198
Lieberman.GM12878	chr12	1,000,000	8,771	8,723	8,680
Lieberman.GM12878	chr12	2,500,000	1,431	1,377	1,331
Lieberman.GM12878	chr13	5,000	235,766	66,668	18,877
Lieberman.GM12878	chr13	10,000	275,004	67,092	63,520
Lieberman.GM12878	chr13	25,000	210,669	65,111	63,015
Lieberman.GM12878	chr13	50,000	171,677	76,351	48,043
Lieberman.GM12878	chr13	100,000	230,125	40,077	38,787
Lieberman.GM12878	chr13	250,000	73,548	67,556	55,952
Lieberman.GM12878	chr13	500,000	18,714	18,630	18,494
Lieberman.GM12878	chr13	1,000,000	4,753	4,710	4,652
Lieberman.GM12878	chr13	2,500,000	820	806	794
Lieberman.GM12878	chr14	5,000	302,032	73,203	67,946
Lieberman.GM12878	chr14	10,000	301,955	82,655	78,171
Lieberman.GM12878	chr14	25,000	212,484	104,436	71,493
Lieberman.GM12878	chr14	50,000	192,226	45,670	44,692
Lieberman.GM12878	chr14	100,000	225,171	48,219	45,773
Lieberman.GM12878	chr14	250,000	59,555	55,615	50,563
Lieberman.GM12878	chr14	500,000	15,534	15,456	15,332
Lieberman.GM12878	chr14	1,000,000	4,000	3,976	3,951
Lieberman.GM12878	chr14	2,500,000	666	666	666
Lieberman.GM12878	chr15	5,000	307,861	79,133	22,267

Continued on next page

Supplementary Table S7 – *Continued from previous page*

**Dataset identifier**	**Chr.**	**Resolution**	**Non-empty blocks**	**IDR < 0.05**	**IDR < 0.01**
Lieberman.GM12878	chr15	10,000	298,024	84,856	79,979
Lieberman.GM12878	chr15	25,000	200,565	100,023	68,502
Lieberman.GM12878	chr15	50,000	182,864	44,409	43,482
Lieberman.GM12878	chr15	100,000	191,179	47,234	44,480
Lieberman.GM12878	chr15	250,000	49,764	45,341	41,843
Lieberman.GM12878	chr15	500,000	13,501	13,381	13,198
Lieberman.GM12878	chr15	1,000,000	3,486	3,474	3,460
Lieberman.GM12878	chr15	2,500,000	562	562	562
Lieberman.GM12878	chr16	5,000	294,568	73,909	66,980
Lieberman.GM12878	chr16	10,000	284,985	77,377	72,133
Lieberman.GM12878	chr16	25,000	191,094	64,648	61,992
Lieberman.GM12878	chr16	50,000	194,390	41,382	40,415
Lieberman.GM12878	chr16	100,000	175,027	55,181	50,542
Lieberman.GM12878	chr16	250,000	46,386	41,366	38,288
Lieberman.GM12878	chr16	500,000	12,858	12,737	12,603
Lieberman.GM12878	chr16	1,000,000	3,320	3,320	3,320
Lieberman.GM12878	chr16	2,500,000	594	589	584
Lieberman.GM12878	chr17	5,000	361,865	92,340	83,640
Lieberman.GM12878	chr17	10,000	313,381	93,428	88,389
Lieberman.GM12878	chr17	25,000	188,238	98,697	77,844
Lieberman.GM12878	chr17	50,000	170,105	39,836	38,882
Lieberman.GM12878	chr17	100,000	180,407	58,239	18,133
Lieberman.GM12878	chr17	250,000	46,859	43,044	39,512
Lieberman.GM12878	chr17	500,000	12,535	12,468	12,363
Lieberman.GM12878	chr17	1,000,000	3,239	3,226	3,212
Lieberman.GM12878	chr17	2,500,000	528	522	514
Lieberman.GM12878	chr18	5,000	211,392	47,932	45,556
Lieberman.GM12878	chr18	10,000	232,114	82,413	27,937
Lieberman.GM12878	chr18	25,000	173,351	90,334	63,470
Lieberman.GM12878	chr18	50,000	162,375	37,368	36,715
Lieberman.GM12878	chr18	100,000	203,727	49,341	45,012
Lieberman.GM12878	chr18	250,000	44,688	43,959	42,626
Lieberman.GM12878	chr18	500,000	11,435	11,350	11,208
Lieberman.GM12878	chr18	1,000,000	3,001	2,981	2,960
Lieberman.GM12878	chr18	2,500,000	528	528	528
Lieberman.GM12878	chr19	5,000	262,686	68,597	61,337
Lieberman.GM12878	chr19	10,000	235,823	69,148	65,221
Lieberman.GM12878	chr19	25,000	151,773	48,195	46,620
Lieberman.GM12878	chr19	50,000	165,471	32,348	31,441
Lieberman.GM12878	chr19	100,000	114,025	68,806	38,232
Lieberman.GM12878	chr19	250,000	24,810	23,787	22,410
Lieberman.GM12878	chr19	500,000	6,508	6,508	6,508

Continued on next page

Supplementary Table S7 – *Continued from previous page*

Dataset identifier	Chr.	Resolution	Non-empty blocks	IDR < 0.05	IDR < 0.01
Lieberman.GM12878	chr19	1,000,000	1,710	1,710	1,710
Lieberman.GM12878	chr19	2,500,000	276	276	276
Lieberman.GM12878	chr20	5,000	248,229	61,439	55,749
Lieberman.GM12878	chr20	10,000	250,620	69,149	64,949
Lieberman.GM12878	chr20	25,000	168,384	89,269	68,188
Lieberman.GM12878	chr20	50,000	173,955	34,616	33,939
Lieberman.GM12878	chr20	100,000	137,648	74,458	36,295
Lieberman.GM12878	chr20	250,000	28,789	28,035	26,798
Lieberman.GM12878	chr20	500,000	7,361	7,353	7,342
Lieberman.GM12878	chr20	1,000,000	1,891	1,891	1,891
Lieberman.GM12878	chr20	2,500,000	351	351	351
Lieberman.GM12878	chr21	5,000	103,984	26,238	6,731
Lieberman.GM12878	chr21	10,000	106,562	29,231	27,199
Lieberman.GM12878	chr21	25,000	82,123	24,613	23,732
Lieberman.GM12878	chr21	50,000	78,571	18,319	17,822
Lieberman.GM12878	chr21	100,000	44,468	23,378	21,431
Lieberman.GM12878	chr21	250,000	9,486	8,682	8,263
Lieberman.GM12878	chr21	500,000	2,616	2,523	2,466
Lieberman.GM12878	chr21	1,000,000	736	728	720
Lieberman.GM12878	chr21	2,500,000	153	152	149
Lieberman.GM12878	chr22	5,000	172,383	45,074	39,721
Lieberman.GM12878	chr22	10,000	158,446	45,952	43,071
Lieberman.GM12878	chr22	25,000	89,777	50,520	39,775
Lieberman.GM12878	chr22	50,000	100,092	18,609	18,340
Lieberman.GM12878	chr22	100,000	49,585	37,930	31,245
Lieberman.GM12878	chr22	250,000	9,333	9,219	9,056
Lieberman.GM12878	chr22	500,000	2,472	2,460	2,440
Lieberman.GM12878	chr22	1,000,000	661	661	661
Lieberman.GM12878	chr22	2,500,000	120	120	120
Lieberman.GM12878	chrX	5,000	269,579	65,015	63,265
Lieberman.GM12878	chrX	10,000	329,318	73,531	70,353
Lieberman.GM12878	chrX	25,000	314,993	86,527	83,587
Lieberman.GM12878	chrX	50,000	333,079	110,587	44,141
Lieberman.GM12878	chrX	100,000	440,210	132,882	39,885
Lieberman.GM12878	chrX	250,000	171,734	103,803	98,227
Lieberman.GM12878	chrX	500,000	46,687	40,843	36,961
Lieberman.GM12878	chrX	1,000,000	11,935	11,543	11,174
Lieberman.GM12878	chrX	2,500,000	2,016	1,954	1,909
Lieberman.Patski	chr1	5,000	32,238	128	3
Lieberman.Patski	chr1	10,000	49,770	3,739	346
Lieberman.Patski	chr1	25,000	57,694	14,445	2,139
Lieberman.Patski	chr1	50,000	46,985	16,202	13,739

Continued on next page

Supplementary Table S7 – *Continued from previous page*

Dataset identifier	Chr.	Resolution	Non-empty blocks	IDR < 0.05	IDR < 0.01
Lieberman.Patski	chr1	100,000	31,015	11,058	10,193
Lieberman.Patski	chr1	250,000	37,635	8,026	7,164
Lieberman.Patski	chr1	500,000	45,687	13,811	9,553
Lieberman.Patski	chr1	1,000,000	17,880	15,392	10,708
Lieberman.Patski	chr1	2,500,000	3,081	3,064	3,025
Lieberman.Patski	chr2	5,000	32,428	133	3
Lieberman.Patski	chr2	10,000	49,666	4,990	495
Lieberman.Patski	chr2	25,000	55,247	19,390	15,514
Lieberman.Patski	chr2	50,000	44,683	15,333	13,364
Lieberman.Patski	chr2	100,000	28,313	13,710	5,902
Lieberman.Patski	chr2	250,000	38,246	7,093	6,404
Lieberman.Patski	chr2	500,000	39,223	14,110	10,364
Lieberman.Patski	chr2	1,000,000	15,077	12,498	9,471
Lieberman.Patski	chr2	2,500,000	2,597	2,594	2,590
Lieberman.Patski	chr3	5,000	52,319	4,386	935
Lieberman.Patski	chr3	10,000	67,860	18,686	11,654
Lieberman.Patski	chr3	25,000	73,569	22,960	17,814
Lieberman.Patski	chr3	50,000	57,542	19,022	16,067
Lieberman.Patski	chr3	100,000	39,804	15,533	4,614
Lieberman.Patski	chr3	250,000	72,703	12,967	10,383
Lieberman.Patski	chr3	500,000	43,092	24,836	9,690
Lieberman.Patski	chr3	1,000,000	12,402	12,230	11,715
Lieberman.Patski	chr3	2,500,000	2,016	2,016	2,016
Lieberman.Patski	chr4	5,000	14,719	42	0
Lieberman.Patski	chr4	10,000	27,131	0	0
Lieberman.Patski	chr4	25,000	31,165	3,260	194
Lieberman.Patski	chr4	50,000	26,064	7,200	921
Lieberman.Patski	chr4	100,000	17,867	6,860	5,696
Lieberman.Patski	chr4	250,000	16,473	4,035	3,619
Lieberman.Patski	chr4	500,000	24,853	4,569	3,303
Lieberman.Patski	chr4	1,000,000	10,507	8,322	5,023
Lieberman.Patski	chr4	2,500,000	1,953	1,941	1,900
Lieberman.Patski	chr5	5,000	40,329	3,891	1,256
Lieberman.Patski	chr5	10,000	52,515	17,060	11,445
Lieberman.Patski	chr5	25,000	54,896	18,485	15,488
Lieberman.Patski	chr5	50,000	42,097	16,245	4,385
Lieberman.Patski	chr5	100,000	29,876	9,495	8,930
Lieberman.Patski	chr5	250,000	51,853	9,891	8,256
Lieberman.Patski	chr5	500,000	35,429	18,231	6,343
Lieberman.Patski	chr5	1,000,000	10,849	10,136	7,696
Lieberman.Patski	chr5	2,500,000	1,830	1,774	1,638
Lieberman.Patski	chr6	5,000	25,092	120	1

Continued on next page

Supplementary Table S7 – *Continued from previous page*

Dataset identifier	Chr.	Resolution	Non-empty blocks	IDR < 0.05	IDR < 0.01
Lieberman.Patski	chr6	10,000	38,470	2,921	300
Lieberman.Patski	chr6	25,000	42,422	10,235	1,500
Lieberman.Patski	chr6	50,000	34,661	11,663	9,718
Lieberman.Patski	chr6	100,000	24,058	8,348	7,279
Lieberman.Patski	chr6	250,000	34,218	6,549	5,558
Lieberman.Patski	chr6	500,000	31,216	8,700	2,361
Lieberman.Patski	chr6	1,000,000	10,738	9,562	5,962
Lieberman.Patski	chr6	2,500,000	1,770	1,753	1,698
Lieberman.Patski	chr7	5,000	24,110	72	0
Lieberman.Patski	chr7	10,000	35,316	9,224	4,988
Lieberman.Patski	chr7	25,000	38,416	9,125	1,184
Lieberman.Patski	chr7	50,000	29,309	10,484	8,740
Lieberman.Patski	chr7	100,000	19,494	7,159	6,456
Lieberman.Patski	chr7	250,000	26,871	5,339	4,483
Lieberman.Patski	chr7	500,000	27,236	7,940	5,585
Lieberman.Patski	chr7	1,000,000	9,250	8,255	4,974
Lieberman.Patski	chr7	2,500,000	1,658	1,641	1,607
Lieberman.Patski	chr8	5,000	24,470	148	8
Lieberman.Patski	chr8	10,000	35,669	9,099	4,823
Lieberman.Patski	chr8	25,000	39,989	9,814	1,466
Lieberman.Patski	chr8	50,000	32,193	11,415	9,705
Lieberman.Patski	chr8	100,000	21,247	7,339	6,681
Lieberman.Patski	chr8	250,000	28,262	5,479	4,885
Lieberman.Patski	chr8	500,000	25,255	8,088	2,332
Lieberman.Patski	chr8	1,000,000	7,896	7,788	7,440
Lieberman.Patski	chr8	2,500,000	1,326	1,326	1,326
Lieberman.Patski	chr9	5,000	25,348	96	4
Lieberman.Patski	chr9	10,000	37,108	11,317	6,311
Lieberman.Patski	chr9	25,000	42,443	11,559	1,691
Lieberman.Patski	chr9	50,000	32,181	11,760	10,179
Lieberman.Patski	chr9	100,000	20,535	9,590	4,223
Lieberman.Patski	chr9	250,000	25,596	4,655	4,359
Lieberman.Patski	chr9	500,000	23,946	9,745	2,988
Lieberman.Patski	chr9	1,000,000	7,458	7,202	6,490
Lieberman.Patski	chr9	2,500,000	1,225	1,193	1,116
Lieberman.Patski	chr10	5,000	22,338	104	2
Lieberman.Patski	chr10	10,000	34,309	4,013	409
Lieberman.Patski	chr10	25,000	40,844	11,524	1,861
Lieberman.Patski	chr10	50,000	33,877	11,382	10,206
Lieberman.Patski	chr10	100,000	21,891	10,667	4,784
Lieberman.Patski	chr10	250,000	25,870	4,527	4,347
Lieberman.Patski	chr10	500,000	25,701	8,273	2,359

Continued on next page

Supplementary Table S7 – *Continued from previous page*

Dataset identifier	Chr.	Resolution	Non-empty blocks	IDR < 0.05	IDR < 0.01
Lieberman.Patski	chr10	1,000,000	8,224	8,016	7,386
Lieberman.Patski	chr10	2,500,000	1,378	1,358	1,311
Lieberman.Patski	chr11	5,000	27,350	66	0
Lieberman.Patski	chr11	10,000	39,517	3,920	312
Lieberman.Patski	chr11	25,000	40,085	14,247	11,471
Lieberman.Patski	chr11	50,000	29,454	11,250	9,906
Lieberman.Patski	chr11	100,000	19,059	9,181	3,706
Lieberman.Patski	chr11	250,000	29,183	5,122	4,549
Lieberman.Patski	chr11	500,000	20,714	10,762	3,743
Lieberman.Patski	chr11	1,000,000	6,836	6,388	5,311
Lieberman.Patski	chr11	2,500,000	1,176	1,176	1,176
Lieberman.Patski	chr12	5,000	13,092	0	0
Lieberman.Patski	chr12	10,000	24,422	423	16
Lieberman.Patski	chr12	25,000	28,186	8,915	6,257
Lieberman.Patski	chr12	50,000	25,070	6,416	959
Lieberman.Patski	chr12	100,000	16,952	6,357	1,460
Lieberman.Patski	chr12	250,000	17,503	2,509	494
Lieberman.Patski	chr12	500,000	19,610	2,863	708
Lieberman.Patski	chr12	1,000,000	6,510	4,679	1,424
Lieberman.Patski	chr12	2,500,000	1,136	1,136	1,136
Lieberman.Patski	chr13	5,000	20,026	191	0
Lieberman.Patski	chr13	10,000	30,135	8,089	3,609
Lieberman.Patski	chr13	25,000	37,466	11,973	9,511
Lieberman.Patski	chr13	50,000	30,298	10,864	9,499
Lieberman.Patski	chr13	100,000	19,848	8,989	3,354
Lieberman.Patski	chr13	250,000	20,516	4,323	4,065
Lieberman.Patski	chr13	500,000	20,506	6,555	1,835
Lieberman.Patski	chr13	1,000,000	6,899	5,988	4,304
Lieberman.Patski	chr13	2,500,000	1,169	1,155	1,123
Lieberman.Patski	chr14	5,000	16,664	0	0
Lieberman.Patski	chr14	10,000	26,991	1,275	91
Lieberman.Patski	chr14	25,000	31,693	9,960	7,529
Lieberman.Patski	chr14	50,000	25,932	7,776	1,324
Lieberman.Patski	chr14	100,000	17,567	5,892	5,198
Lieberman.Patski	chr14	250,000	21,956	4,491	3,934
Lieberman.Patski	chr14	500,000	21,556	5,393	1,515
Lieberman.Patski	chr14	1,000,000	6,908	6,571	5,166
Lieberman.Patski	chr14	2,500,000	1,197	1,178	1,153
Lieberman.Patski	chr15	5,000	20,456	95	1
Lieberman.Patski	chr15	10,000	30,156	7,855	4,317
Lieberman.Patski	chr15	25,000	33,659	11,134	8,925
Lieberman.Patski	chr15	50,000	26,573	9,281	7,963

Continued on next page

Supplementary Table S7 – *Continued from previous page*

**Dataset identifier**	**Chr.**	**Resolution**	**Non-empty blocks**	**IDR < 0.05**	**IDR < 0.01**
Lieberman.Patski	chr15	100,000	17,898	6,117	5,752
Lieberman.Patski	chr15	250,000	24,739	4,884	4,334
Lieberman.Patski	chr15	500,000	17,505	6,958	2,231
Lieberman.Patski	chr15	1,000,000	5,090	4,765	3,294
Lieberman.Patski	chr15	2,500,000	861	859	852
Lieberman.Patski	chr16	5,000	15,798	55	0
Lieberman.Patski	chr16	10,000	24,796	6,322	3,392
Lieberman.Patski	chr16	25,000	28,585	9,955	7,757
Lieberman.Patski	chr16	50,000	23,335	8,218	7,321
Lieberman.Patski	chr16	100,000	16,185	5,383	5,074
Lieberman.Patski	chr16	250,000	22,867	4,020	3,731
Lieberman.Patski	chr16	500,000	15,875	9,069	3,542
Lieberman.Patski	chr16	1,000,000	4,575	4,336	3,504
Lieberman.Patski	chr16	2,500,000	780	772	757
Lieberman.Patski	chr17	5,000	17,464	29	0
Lieberman.Patski	chr17	10,000	25,690	2,435	154
Lieberman.Patski	chr17	25,000	27,525	9,512	7,301
Lieberman.Patski	chr17	50,000	22,758	7,580	1,501
Lieberman.Patski	chr17	100,000	14,732	5,309	4,719
Lieberman.Patski	chr17	250,000	20,904	3,723	3,067
Lieberman.Patski	chr17	500,000	14,104	4,926	1,374
Lieberman.Patski	chr17	1,000,000	4,247	3,661	965
Lieberman.Patski	chr17	2,500,000	703	638	429
Lieberman.Patski	chr18	5,000	15,706	77	6
Lieberman.Patski	chr18	10,000	24,024	6,515	3,560
Lieberman.Patski	chr18	25,000	28,112	9,656	7,793
Lieberman.Patski	chr18	50,000	23,350	8,009	6,986
Lieberman.Patski	chr18	100,000	14,953	7,321	3,227
Lieberman.Patski	chr18	250,000	22,521	4,087	3,649
Lieberman.Patski	chr18	500,000	14,287	8,634	3,658
Lieberman.Patski	chr18	1,000,000	3,915	3,906	3,886
Lieberman.Patski	chr18	2,500,000	666	656	631
Lieberman.Patski	chr19	5,000	14,235	91	4
Lieberman.Patski	chr19	10,000	19,920	6,578	3,939
Lieberman.Patski	chr19	25,000	21,167	7,232	6,023
Lieberman.Patski	chr19	50,000	15,252	5,691	5,115
Lieberman.Patski	chr19	100,000	9,893	4,744	2,478
Lieberman.Patski	chr19	250,000	15,083	2,778	2,348
Lieberman.Patski	chr19	500,000	6,585	3,586	1,417
Lieberman.Patski	chr19	1,000,000	1,768	1,368	407
Lieberman.Patski	chr19	2,500,000	300	286	244
Lieberman.Patski	chrX	5,000	9,548	0	0

Continued on next page

Supplementary Table S7 – *Continued from previous page*

Dataset identifier	Chr.	Resolution	Non-empty blocks	IDR < 0.05	IDR < 0.01
Lieberman.Patski	chrX	10,000	20,875	506	47
Lieberman.Patski	chrX	25,000	21,528	2,182	278
Lieberman.Patski	chrX	50,000	21,134	6,041	4,532
Lieberman.Patski	chrX	100,000	18,567	6,090	4,840
Lieberman.Patski	chrX	250,000	26,115	5,461	4,533
Lieberman.Patski	chrX	500,000	37,066	4,580	3,305
Lieberman.Patski	chrX	1,000,000	11,361	2,002	1,216
Lieberman.Patski	chrX	2,500,000	2,147	1,889	498
Skok.NSD2	chr1	5,000	7,183	324	146
Skok.NSD2	chr1	10,000	59,428	2,586	422
Skok.NSD2	chr1	25,000	104,544	34,476	28,899
Skok.NSD2	chr1	50,000	85,480	27,070	25,441
Skok.NSD2	chr1	100,000	57,925	26,805	15,134
Skok.NSD2	chr1	250,000	56,502	10,362	2,946
Skok.NSD2	chr1	500,000	35,766	14,086	11,650
Skok.NSD2	chr1	1,000,000	16,333	9,140	8,741
Skok.NSD2	chr1	2,500,000	4,382	3,290	2,540
Skok.NSD2	chr2	5,000	369	10	0
Skok.NSD2	chr2	10,000	36,839	1,478	659
Skok.NSD2	chr2	25,000	98,219	33,740	28,302
Skok.NSD2	chr2	50,000	91,055	27,868	25,774
Skok.NSD2	chr2	100,000	57,322	30,194	21,454
Skok.NSD2	chr2	250,000	49,081	9,775	9,458
Skok.NSD2	chr2	500,000	47,012	14,497	11,524
Skok.NSD2	chr2	1,000,000	24,521	15,432	11,299
Skok.NSD2	chr2	2,500,000	4,753	4,446	3,917
Skok.NSD2	chr3	5,000	189	109	0
Skok.NSD2	chr3	10,000	24,488	681	284
Skok.NSD2	chr3	25,000	73,880	19,835	3,508
Skok.NSD2	chr3	50,000	70,204	22,088	20,317
Skok.NSD2	chr3	100,000	45,350	22,624	13,028
Skok.NSD2	chr3	250,000	38,716	7,441	7,252
Skok.NSD2	chr3	500,000	33,083	10,380	8,102
Skok.NSD2	chr3	1,000,000	16,338	8,794	7,981
Skok.NSD2	chr3	2,500,000	3,231	2,387	2,142
Skok.NSD2	chr4	5,000	147	124	19
Skok.NSD2	chr4	10,000	27,788	832	378
Skok.NSD2	chr4	25,000	78,558	26,161	21,253
Skok.NSD2	chr4	50,000	77,172	22,768	20,844
Skok.NSD2	chr4	100,000	49,559	17,749	16,812
Skok.NSD2	chr4	250,000	44,461	8,918	8,594
Skok.NSD2	chr4	500,000	36,151	11,155	3,227

Continued on next page

Supplementary Table S7 – *Continued from previous page*

Dataset identifier	Chr.	Resolution	Non-empty blocks	IDR < 0.05	IDR < 0.01
Skok.NSD2	chr4	1,000,000	14,713	10,762	8,991
Skok.NSD2	chr4	2,500,000	2,946	2,566	2,222
Skok.NSD2	chr5	5,000	185	0	0
Skok.NSD2	chr5	10,000	25,695	684	307
Skok.NSD2	chr5	25,000	74,824	25,411	20,550
Skok.NSD2	chr5	50,000	71,639	23,117	20,737
Skok.NSD2	chr5	100,000	45,220	24,230	17,046
Skok.NSD2	chr5	250,000	35,620	7,628	7,413
Skok.NSD2	chr5	500,000	32,006	9,835	7,855
Skok.NSD2	chr5	1,000,000	14,541	9,831	7,296
Skok.NSD2	chr5	2,500,000	2,640	2,529	2,322
Skok.NSD2	chr6	5,000	57	52	47
Skok.NSD2	chr6	10,000	13,274	602	380
Skok.NSD2	chr6	25,000	53,999	19,303	14,980
Skok.NSD2	chr6	50,000	55,872	16,844	15,421
Skok.NSD2	chr6	100,000	35,996	12,697	12,029
Skok.NSD2	chr6	250,000	26,805	6,028	5,886
Skok.NSD2	chr6	500,000	23,632	6,469	5,457
Skok.NSD2	chr6	1,000,000	11,825	6,490	6,062
Skok.NSD2	chr6	2,500,000	2,414	2,090	1,596
Skok.NSD2	chr7	5,000	511	21	0
Skok.NSD2	chr7	10,000	35,545	104	0
Skok.NSD2	chr7	25,000	78,273	25,909	22,158
Skok.NSD2	chr7	50,000	67,147	22,188	20,377
Skok.NSD2	chr7	100,000	41,681	21,105	13,848
Skok.NSD2	chr7	250,000	40,967	7,832	7,433
Skok.NSD2	chr7	500,000	29,883	10,522	8,104
Skok.NSD2	chr7	1,000,000	12,203	7,217	5,995
Skok.NSD2	chr7	2,500,000	2,079	1,958	1,643
Skok.NSD2	chr8	5,000	418	4	0
Skok.NSD2	chr8	10,000	24,591	894	407
Skok.NSD2	chr8	25,000	63,627	21,560	17,580
Skok.NSD2	chr8	50,000	58,076	19,876	17,854
Skok.NSD2	chr8	100,000	36,960	13,713	12,964
Skok.NSD2	chr8	250,000	32,135	6,724	6,493
Skok.NSD2	chr8	500,000	26,902	8,680	2,510
Skok.NSD2	chr8	1,000,000	10,121	6,866	5,558
Skok.NSD2	chr8	2,500,000	1,721	1,635	1,454
Skok.NSD2	chr9	5,000	95	4	0
Skok.NSD2	chr9	10,000	18,667	430	192
Skok.NSD2	chr9	25,000	47,214	17,042	13,965
Skok.NSD2	chr9	50,000	44,072	15,894	3,803

Continued on next page

Supplementary Table S7 – *Continued from previous page*

**Dataset identifier**	**Chr.**	**Resolution**	**Non-empty blocks**	**IDR < 0.05**	**IDR < 0.01**
Skok.NSD2	chr9	100,000	27,379	10,560	9,873
Skok.NSD2	chr9	250,000	22,980	4,569	4,403
Skok.NSD2	chr9	500,000	13,514	6,081	2,221
Skok.NSD2	chr9	1,000,000	6,356	4,035	2,703
Skok.NSD2	chr9	2,500,000	1,252	1,162	1,008
Skok.NSD2	chr10	5,000	95	65	0
Skok.NSD2	chr10	10,000	19,750	642	337
Skok.NSD2	chr10	25,000	54,909	15,644	2,380
Skok.NSD2	chr10	50,000	49,513	21,305	7,411
Skok.NSD2	chr10	100,000	32,416	15,834	9,450
Skok.NSD2	chr10	250,000	27,151	5,668	5,464
Skok.NSD2	chr10	500,000	21,014	6,680	5,790
Skok.NSD2	chr10	1,000,000	8,651	5,976	4,531
Skok.NSD2	chr10	2,500,000	1,485	1,334	994
Skok.NSD2	chr11	5,000	199	38	0
Skok.NSD2	chr11	10,000	15,021	496	254
Skok.NSD2	chr11	25,000	45,399	16,522	13,331
Skok.NSD2	chr11	50,000	42,376	16,993	5,256
Skok.NSD2	chr11	100,000	28,023	13,293	6,520
Skok.NSD2	chr11	250,000	22,134	4,763	4,604
Skok.NSD2	chr11	500,000	16,807	4,774	4,107
Skok.NSD2	chr11	1,000,000	6,159	4,933	4,177
Skok.NSD2	chr11	2,500,000	1,504	1,096	1,076
Skok.NSD2	chr12	5,000	2,079	68	29
Skok.NSD2	chr12	10,000	24,070	3,065	1,994
Skok.NSD2	chr12	25,000	56,341	18,753	14,732
Skok.NSD2	chr12	50,000	49,026	18,592	5,094
Skok.NSD2	chr12	100,000	33,582	14,341	6,154
Skok.NSD2	chr12	250,000	24,672	6,201	5,817
Skok.NSD2	chr12	500,000	20,059	4,916	1,333
Skok.NSD2	chr12	1,000,000	7,375	5,353	4,045
Skok.NSD2	chr12	2,500,000	1,462	1,422	1,339
Skok.NSD2	chr13	10,000	823	6	0
Skok.NSD2	chr13	25,000	20,749	4,916	526
Skok.NSD2	chr13	50,000	25,725	8,562	7,490
Skok.NSD2	chr13	100,000	18,259	7,401	6,956
Skok.NSD2	chr13	250,000	10,826	4,734	2,606
Skok.NSD2	chr13	500,000	11,534	2,407	2,151
Skok.NSD2	chr13	1,000,000	4,629	2,628	1,233
Skok.NSD2	chr13	2,500,000	782	665	384
Skok.NSD2	chr14	5,000	46	46	46
Skok.NSD2	chr14	10,000	6,832	162	82

Continued on next page

Supplementary Table S7 – *Continued from previous page*

Dataset identifier	Chr.	Resolution	Non-empty blocks	IDR < 0.05	IDR < 0.01
Skok.NSD2	chr14	25,000	27,449	9,924	7,765
Skok.NSD2	chr14	50,000	26,681	8,389	7,788
Skok.NSD2	chr14	100,000	18,180	8,647	4,796
Skok.NSD2	chr14	250,000	11,792	4,404	2,216
Skok.NSD2	chr14	500,000	11,121	2,526	2,273
Skok.NSD2	chr14	1,000,000	3,781	3,116	1,835
Skok.NSD2	chr14	2,500,000	665	637	584
Skok.NSD2	chr15	10,000	11,170	448	256
Skok.NSD2	chr15	25,000	30,972	11,450	9,627
Skok.NSD2	chr15	50,000	27,323	9,318	8,682
Skok.NSD2	chr15	100,000	17,367	8,771	5,895
Skok.NSD2	chr15	250,000	12,969	2,863	2,777
Skok.NSD2	chr15	500,000	10,173	2,556	2,240
Skok.NSD2	chr15	1,000,000	3,329	2,995	2,054
Skok.NSD2	chr15	2,500,000	574	574	574
Skok.NSD2	chr16	5,000	402	25	15
Skok.NSD2	chr16	10,000	17,962	1,069	452
Skok.NSD2	chr16	25,000	33,544	11,167	2,069
Skok.NSD2	chr16	50,000	28,227	12,669	4,876
Skok.NSD2	chr16	100,000	18,720	8,946	4,969
Skok.NSD2	chr16	250,000	14,443	3,629	3,419
Skok.NSD2	chr16	500,000	9,171	2,667	2,428
Skok.NSD2	chr16	1,000,000	3,184	2,597	2,115
Skok.NSD2	chr16	2,500,000	582	580	577
Skok.NSD2	chr17	5,000	200	25	0
Skok.NSD2	chr17	10,000	15,381	586	236
Skok.NSD2	chr17	25,000	28,274	9,523	1,440
Skok.NSD2	chr17	50,000	22,159	10,859	3,957
Skok.NSD2	chr17	100,000	13,581	7,498	5,813
Skok.NSD2	chr17	250,000	11,035	2,133	2,064
Skok.NSD2	chr17	500,000	8,360	2,684	2,100
Skok.NSD2	chr17	1,000,000	3,031	2,437	1,603
Skok.NSD2	chr17	2,500,000	557	557	557
Skok.NSD2	chr18	5,000	287	2	0
Skok.NSD2	chr18	10,000	15,712	921	392
Skok.NSD2	chr18	25,000	36,877	12,341	10,286
Skok.NSD2	chr18	50,000	32,173	10,657	9,738
Skok.NSD2	chr18	100,000	20,509	9,811	5,015
Skok.NSD2	chr18	250,000	17,938	3,904	3,694
Skok.NSD2	chr18	500,000	10,243	3,408	2,947
Skok.NSD2	chr18	1,000,000	2,807	2,586	1,691
Skok.NSD2	chr18	2,500,000	507	493	462

Continued on next page

Supplementary Table S7 – *Continued from previous page*

Dataset identifier	Chr.	Resolution	Non-empty blocks	IDR < 0.05	IDR < 0.01
Skok.NSD2	chr19	5,000	277	43	0
Skok.NSD2	chr19	10,000	13,189	161	12
Skok.NSD2	chr19	25,000	24,387	8,309	1,586
Skok.NSD2	chr19	50,000	20,006	10,060	6,022
Skok.NSD2	chr19	100,000	14,277	4,358	4,233
Skok.NSD2	chr19	250,000	12,180	2,532	2,399
Skok.NSD2	chr19	500,000	5,627	3,490	1,946
Skok.NSD2	chr19	1,000,000	1,650	1,592	1,471
Skok.NSD2	chr19	2,500,000	298	298	298
Skok.NSD2	chr20	5,000	267	0	0
Skok.NSD2	chr20	10,000	12,988	143	12
Skok.NSD2	chr20	25,000	26,001	8,874	7,561
Skok.NSD2	chr20	50,000	21,922	9,938	4,225
Skok.NSD2	chr20	100,000	14,783	6,789	4,033
Skok.NSD2	chr20	250,000	10,476	2,396	2,242
Skok.NSD2	chr20	500,000	6,438	2,315	2,144
Skok.NSD2	chr20	1,000,000	2,000	1,673	1,164
Skok.NSD2	chr20	2,500,000	351	326	268
Skok.NSD2	chr21	5,000	56	56	56
Skok.NSD2	chr21	10,000	7,385	192	78
Skok.NSD2	chr21	25,000	16,158	5,641	4,748
Skok.NSD2	chr21	50,000	14,240	4,842	4,417
Skok.NSD2	chr21	100,000	9,131	4,749	2,951
Skok.NSD2	chr21	250,000	6,552	1,402	1,362
Skok.NSD2	chr21	500,000	2,431	1,927	1,561
Skok.NSD2	chr21	1,000,000	699	658	612
Skok.NSD2	chr21	2,500,000	153	129	122
Skok.NSD2	chr22	5,000	26	26	26
Skok.NSD2	chr22	10,000	6,104	109	41
Skok.NSD2	chr22	25,000	13,006	5,129	4,450
Skok.NSD2	chr22	50,000	10,860	5,022	2,071
Skok.NSD2	chr22	100,000	6,471	3,523	2,817
Skok.NSD2	chr22	250,000	5,325	1,302	1,152
Skok.NSD2	chr22	500,000	2,277	1,505	930
Skok.NSD2	chr22	1,000,000	673	642	589
Skok.NSD2	chr22	2,500,000	141	139	136
Skok.NSD2	chrX	10,000	4,310	3	0
Skok.NSD2	chrX	25,000	34,214	7,574	987
Skok.NSD2	chrX	50,000	42,908	13,666	11,329
Skok.NSD2	chrX	100,000	32,173	14,589	5,103
Skok.NSD2	chrX	250,000	27,555	6,623	6,346
Skok.NSD2	chrX	500,000	19,387	6,640	6,043

Continued on next page

Supplementary Table S7 – *Continued from previous page*

**Dataset identifier**	**Chr.**	**Resolution**	**Non-empty blocks**	**IDR < 0.05**	**IDR < 0.01**
Skok.NSD2	chrX	1,000,000	9,628	4,364	4,182
Skok.NSD2	chrX	2,500,000	1,957	1,418	969

Supplementary Table S7: IDR2D analysis of individual chromosomes in three pairs of HiC experiments. Columns are (1) *chromosome identifier*, (2) *block resolution in base pairs*, (3) *number of non-empty (20 or more reads) blocks*, (4) *number of reproducible blocks with IDR* < 0.05, and (5) *number of reproducible blocks with IDR* < 0.01.

References

[1] Qunhua Li, James B. Brown, Haiyan Huang, and Peter J. Bickel. Measuring reproducibility of high-throughput experiments. *Ann. Appl. Stat.*, 5(3):1752–1779, 09 2011.
